# Using stable isotopes in hummingbird breath to estimate reliance on supplemental feeders

**DOI:** 10.1002/ece3.9799

**Published:** 2023-02-07

**Authors:** Nathan Wolf, T. Scott Smeltz, Craig Cook, Carlos Martinez del Rio

**Affiliations:** ^1^ FAST Laboratory Alaska Pacific University Anchorage Alaska USA; ^2^ University of Wyoming Stable Isotope Facility, University of Wyoming Laramie Wyoming USA; ^3^ Department of Zoology and Physiology University of Wyoming Laramie Wyoming USA

**Keywords:** breath, feeder, hummingbird, pollinator, stable isotopes, δ^13^C

## Abstract

Understanding the ecological consequences of supplemental feeding to both hummingbirds and the plants they pollinate is complicated by logistical challenges associated with assessing relative dietary resource use with commonly applied observational methods. Here, we describe the results of research conducted to assess the relative use of feeder and flower nectar by Broad‐tailed (*Selasphorus platycercus*) and Rufous hummingbirds (*Selasphorus rufus*) using two distinct methodological variations to measure the δ^13^C values of exhaled CO_2_. Because of the relatively quick time in which both species switch from exogenous to endogenous resources to fuel metabolism, our experiment allowed us to assess resource use at two timescales. Our results suggest variability in the relative contributions of the two dietary sources within and among species and timescales, with most birds employing a mixture of feeder and flower sugars as fuel sources. This diversity in relative resource use may mitigate potential negative effects of supplemental feeding on hummingbirds and their plant symbionts.

## Graphical abstract

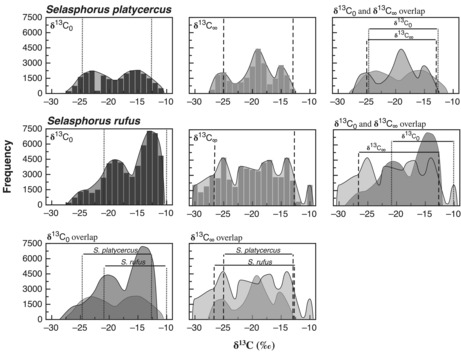

## 1 INTRODUCTION

Humans often supplement the diets of wild birds with feeders (Jones, 2018), a practice that has been shown to affect body condition, survival, and reproduction (Ruffino et al., 2014), winter range expansion (Greig et al., 2017), and the local composition of bird assemblages (Galbraith et al., 2015). In the Americas, the use of hummingbird feeders has increased enormously both as a recreational activity and in concert with the proliferation of eco‐tourism opportunities that cater to birdwatchers (Ridgely, 2011). With few exceptions (see Greig et al., 2017), the consequences of this increase in supplemental feeding on both hummingbird populations and the flowers they pollinate remain unknown, and there is a largely unresolved debate on whether the presence of feeders has a positive or negative influence on hummingbirds' role as pollinators (Arizmendi et al., 2007; Sonne et al., 2016). Previous studies suggest an inverse relationship between feeder visitation rates and local floral abundances (Inouye et al., 1991; McCafferey & Wethington, 2008). However, data that estimates the relative use of feeders and flowers by individual hummingbirds is both limited and instrumental to assessing the impact of feeders on hummingbird dietary ecology and consequent flower pollination. Unfortunately, assessing relative dietary resource of individual birds use with traditional techniques, such as direct observation, is logistically challenging. Here, we describe a relatively simple method to quantify the contribution of feeders to the energy budgets of hummingbirds at both individual and population scales with the potential for multiple temporal scales of inference.

Our method is an extension of approaches used to measure the carbon‐isotope composition of CO_2_ in exhaled breath (Welch et al., 2016). Briefly, in fed hummingbirds, the carbon stable isotope (δ^13^C) composition of exhaled CO_2_ closely reflects recently consumed sugars. Conversely, in fasted birds, the δ^13^C of exhaled CO_2_ reflects the sugars used to synthesize lipid reserves minus a small depletion (~0.6%–2.9%; Carleton et al., 2004, 2006) caused during lipid synthesis. While δ^13^C analysis of exhaled CO_2_ does not allow for identification of specific endogenous substrates metabolized by fasted hummingbirds, previous studies using respiratory quotient suggest that fasted hummingbirds oxidize primarily lipids (Carleton et al., 2006; Powers, 1991; Suarez et al., 1990; Suarez & Gass, 2002 and references therein). Further, the small depletion in the δ^13^C of exhaled CO_2_ in fasted birds observed by Carleton et al. (2004, 2006) is in keeping with expectations of the relative difference in δ^13^C between carbohydrates and lipids (Deniro & Epstein, 1977). Carleton et al. (2006) estimated that the average residence time of a carbon molecule in the lipid reserves of Broad‐tailed hummingbirds (*Selasphorus platycercus*) ranges from 1 to 2 days. However, this estimate hinges on the size of lipid reserves (average residence time equals the ratio of size of reserves/input rate). Further, the time required for an individual bird to switch from using recently consumed sugars to lipid reserves varies according to the size and timing of recent meals and consequent crop fullness. Karasov et al. (1986) found that crop‐emptying times for captive Rufous hummingbirds (*Selasphorus rufus*) ranged between 15 and 20 min with a full passage rate of ≤15 min. Accordingly, for these species, we can qualitatively assume (1) for fed individuals, the δ^13^C value of breath reflects the value of sugars ingested on a time scale of minutes, (2) individuals can be considered fasted on a time scale of minutes to tens of minutes, and (3) the δ^13^C value of breath in a fasted hummingbird reflects the δ^13^C value of sugars ingested and integrated into lipid reserves over a time scale of days. By conducting measurements of δ^13^C in exhaled CO_2_ on timescales longer than those required for crop emptying/passage (i.e., in fed and fasted birds), we can characterize the use of dietary sugars at two time scales: the last few minutes in fed birds and the last few days in fasted birds.

Using this technique to determine the relative contribution of feeders to the diets of wild hummingbirds relies on the contrasting values of the sugars synthesized by plants using different photosynthetic pathways. Plants that rely on C_3_ photosynthesis synthesize sugars that are more depleted in ^13^C, the heavy isotope of carbon, than plants that use C_4_ and CAM photosynthesis (Bender, 1971). As a result, the δ^13^C values of C_4_ sugars range from −18‰ to −8‰, whereas those of C_3_ and CAM plants range from −35‰ to −20‰ (O'Leary, 1988). Many CAM plants can; however, shift facultatively between CAM and C_3_ photosynthesis and their δ^13^C values vary according to the dominant pathway (Winter & Holtum, 2002). In many, albeit not all ecosystems, the plants pollinated by hummingbirds use a single photosynthetic pathway. For example, in the Rocky Mountains of North America, all plants pollinated by hummingbirds use C_3_ photosynthesis (Still et al., 2003). In other ecosystems, such as arid deserts, the most abundant nectar sources can be CAM plants (Wolf & Hatch, 2011). By furnishing feeders with sucrose solutions manufactured from plants with a different photosynthetic pathway from that dominant in an ecosystem (e.g., C_3_ beet sugar or C_4_ cane sugar), we can quantify the relative contribution of feeders and flowers to the energy budgets of hummingbirds.

We used the δ^13^C value of exhaled CO_2_ to assess the relative contributions of feeders and flowers to the energy budgets of Broad‐tailed and Rufous hummingbirds during two summers, at two different sites in the Rocky Mountains of North America. Further, we used two methodological variations to assess the δ^13^C of exhaled CO_2_. One methodological variation represents a significant technical advance over the other but requires significantly more equipment. This second, more analytically advanced effort stemmed from our inability to interpret the results of our first effort clearly.

## 2 METHODS

The work described in this article was approved by the University of Wyoming's Institutional Animal Care and Use Committee (#A‐3216‐01 and #20180403CM00303‐01 for 2010 and 2018, respectively) and conducted under United States Fish and Wildlife Service permit numbers MB213019‐0 for 2010 and MB84888C‐0 for 2018 and Wyoming Game and Fish Department Chapter 33 Permit numbers 708 for 2010 and 1170 for 2018.

**FIGURE 1:**
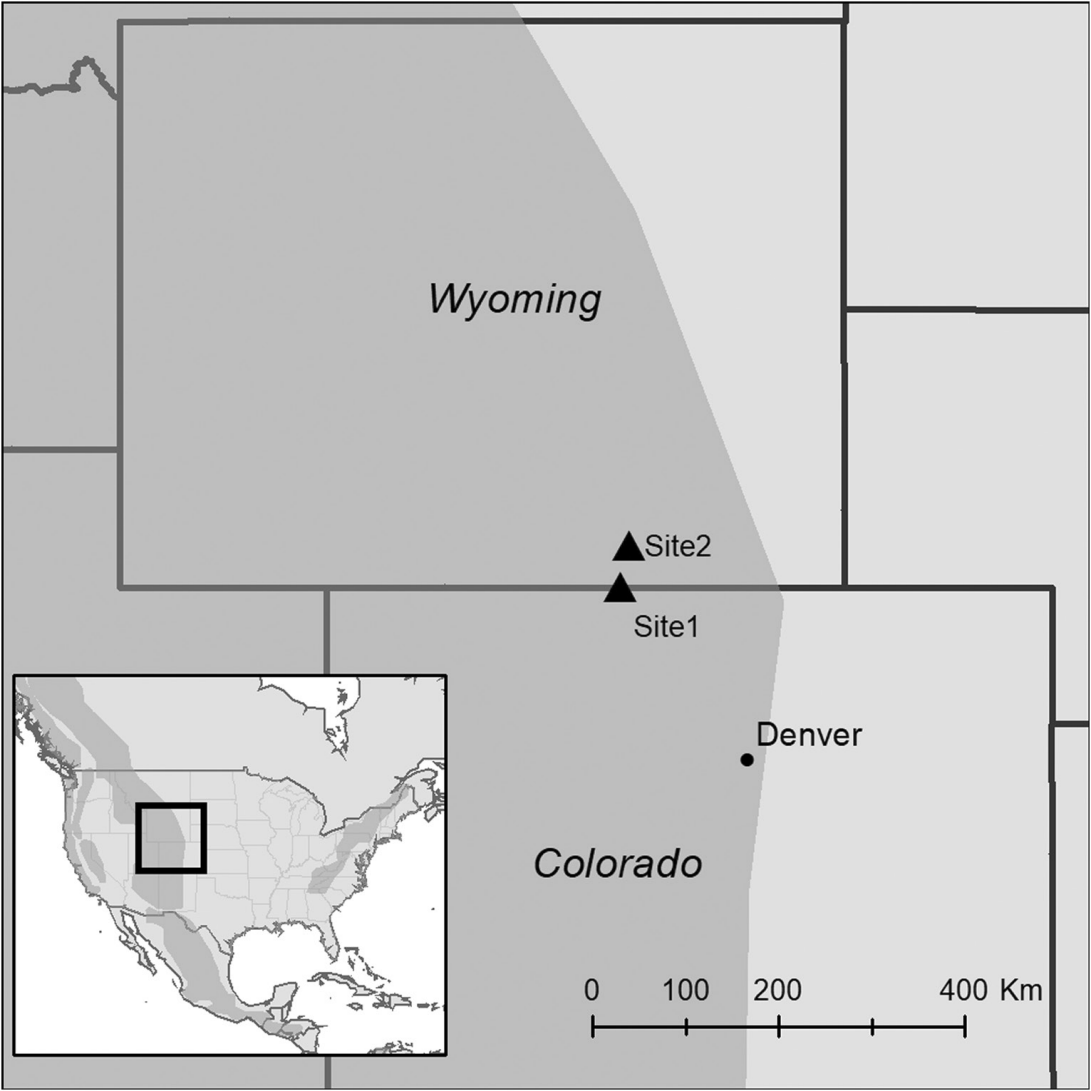
Field sites for 2010 (Site 1) and 2018 (Site 2) data collection efforts were located in southeastern Wyoming. In addition to hosting ample numbers of Broad‐tailed and Rufous hummingbirds in the summer months, all local plants species visited by hummingbirds at these sites use C_3_ photosynthesis, and their foliage varies relatively little in isotopic value. Major topographical features, including the Rocky Mountains, are represented in grey shading.

First methodological variation—On June 7, 2010, we placed four hummingbird feeders containing C_4_ cane sugar nectar (25% weight/volume, δ^13^C average ± sd = −11.4‰ ± 0.1‰; this concentration is recommended for hummingbird feeders and is similar to that of many hummingbird‐pollinated flower species; Baker, 1975) at a remote location near Wycolo in the Snowy Mountains of Wyoming, USA (41° 00′ 25.25″ N, 106° 12′ 49.38″ W, elevation 2644 m; Figure 1). All local plants species visited by hummingbirds at this site (e.g., *Penstemon cyaneus, Epilobium angustifolium, Castilleja sulphurea, and Castilleja linarifolia*) use C_3_ photosynthesis, and their flowers show low overall variation in bulk δ^13^C values (*n* = 18, δ^13^C average ± sd = −26.6‰ ± 1.1‰). While previous work on C_3_ plants (*Brassica napus L*.) has shown that nectar is slightly enriched in δ^13^C compared to flowers (≤1‰; Hongxia et al., 2022), we chose to use the flower values in our analyses because of the interindividual variability shown in Hongxia et al. (2022) results and the relatively small value of the enrichment relative to the variability in δ^13^C values of inflorescences collected for our study. We recognize; however, that while negligible for our study, this enrichment and the variability among inflorescences may be an important consideration for groups attempting to apply similar methods to investigate other topics (e.g., relative use of nectars from different flower species).

One week after deploying the feeders, we began trapping birds using a drop net trap (Ruschi, 2009). For each captured bird, we determined species and sex by plumage inspection, and we collected a series of five breath samples at 0 (immediately after the bird was removed from the trap), 3, 6, 9, and 12 min. In the wild, hummingbirds feed in distinct bouts (14–18 per hour; Karasov et al., 1986). Between bouts, hummingbirds rest and allow their crop to partially empty before moving to acquire additional nectar (Tiebout, 1989 and references there). Because of this bout‐feeding strategy, and based on the crop emptying time provided by Karasov et al. (1986), we assumed that captured birds' crops would be partially, but not completely, full and that 12 min would provide sufficient time for birds to switch from recently ingested sugars to endogenous (stored) lipid sources. All the breath samples were collected using the Carleton et al. (2006) protocol. Briefly, birds were lightly restrained with a paper sleeve and placed in a 50‐ml polypropylene centrifuge tube. After introducing the bird into the tube, we flushed the tube with CO_2_‐free air for 15 s (approximately 250 ml). The tube was then sealed, and exhaled CO_2_ was allowed to accumulate for 45 s. A 30 ml air sample was then extracted using a gastight syringe. We immediately repressurized the tube following the sample extraction by opening a stop‐cock valve fitted to the tube. Air samples were injected into pre‐evacuated gastight vials (Exetainer; Labco Ltd. Buckinghamshire, UK). This procedure was repeated for each sample. Prior to the release of each bird, we clipped a unique pattern of tail feathers for future identification in the event of recapture. Between June 15 and August 3, 2010, we captured 13 individual Broad‐tailed hummingbirds (5 males and 8 females) and 13 Rufous hummingbirds (13 females).

δ^13^C values for breath samples were measured on a Thermo Finnegan Delta+XP continuous flow mass spectrometer (Thermo Scientific, Bremen, Germany) coupled to a Thermo Finnegan GasBench II (Thermo Scientific, Bremen, Germany) at the Light Stable Isotope Facility, University of Wyoming (Laramie, WY, USA). Replicate analyses of three reference standards (UWSIF 203, δ^13^C = −2.89‰; UWSIF 204, δ^13^C = −10.55‰; and UWSIF 208, δ^13^C = −24.21‰) were run throughout the sample analyses and yielded a precision (standard deviation) of 0.3‰. Carbon stable isotope ratios were expressed using standard delta notation (δ^13^C) in parts per million (‰) as:

$$\delta^{13}C=\left(R_{\text{sample}}∕R_{\text{standard}}\right)*1000,$$

where R_sample_ and R_standard_ are ^13^C / ^12^C of the sample and a standard reference material, in this case, Vienna Pee Dee Belemnite (VPDB).

Second methodological variation—On July 21, 2018, we placed eight hummingbird feeders containing C_4_ cane sugar nectar (25% weight/volume, δ^13^C average ± sd = −12.2 ± 0.3‰) at a location near the town of Centennial at the foot of the Snowy Mountains of Wyoming, USA (41° 18′ 16.24″ N, 106° 07′ 32.48″ W, elevation 2473 m; Figure 1). Given that the hummingbirds at site 2 visited the same plant species as those at site 1 (e.g., *Penstemon cyaneus*, *Epilobium angustifolium*, *Castilleja sulphurea*, *and Castilleja linarifolia*), and the strong similarities in habitat and weather patterns between the two sites/years, we assumed limited differences in δ^13^C values of floral nectar between sites (Novotny & Goodell, 2022) and applied the flower δ^13^C values collected at site 1 in 2010 to all analyses (*n* = 18, δ^13^C average ± sd = −26.6‰ ± 1.1‰).

**FIGURE 2:**
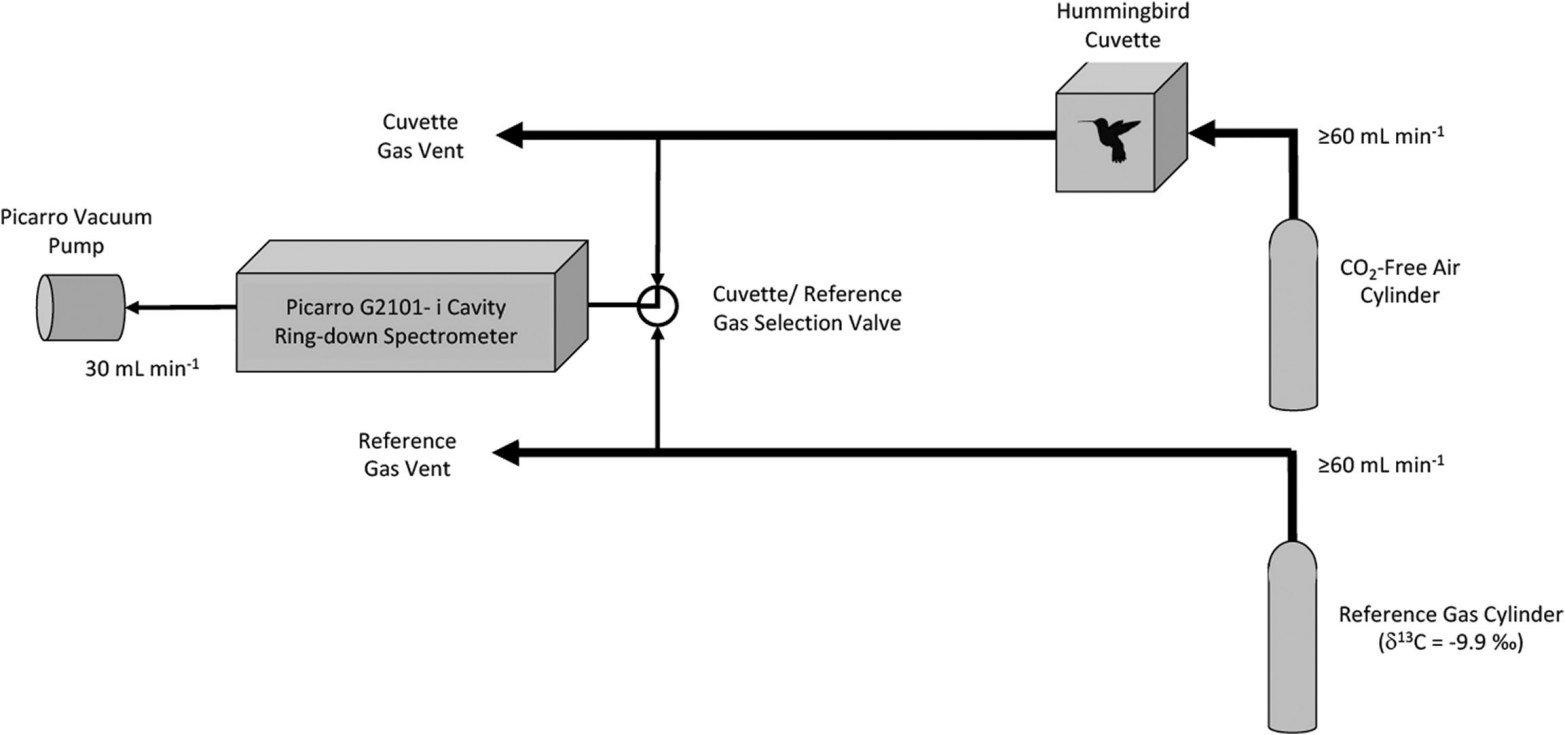
In 2018, δ^13^C values of exhaled CO_2_ were measured by placing hummingbirds in a polypropylene cuvette attached to a Picarro G2101‐ i Cavity Ring‐down Spectrometer designed to measure both the concentration and the δ^13^C value of CO_2_ in air. The cuvette was continuously flushed with CO_2_‐free air at a rate ≥60 ml min^−1^. We ensured cuvette equilibration with CO_2_‐free air before beginning measurements of exhaled CO_2_ by monitoring CO_2_ concentration.

Two days after deploying the feeders, we began trapping birds using a combination of drop net traps (Ruschi, 2009) and mist nets placed in a grid around the feeders. All traps and nets were monitored continuously from daybreak to dusk, and birds were removed promptly upon capture. Between July 23 and July 25, 2018, we captured 3 Broad‐tailed hummingbirds (1 male and 2 females) and 21 Rufous hummingbirds (8 males and 13 females). Differences in both the time period between which feeders were initially deployed and trapping started and in the total number of days for which trapping occurred between the 2010 and 2018 field efforts resulted from logistical constraints in 2018. Immediately after removal, species and sex were determined by plumage inspection, and birds were lightly restrained in a small mesh bag and placed in a polypropylene cuvette attached to a Picarro G2101‐ i Cavity Ring‐down Spectrometer designed to measure both the concentration and the δ^13^C value of CO_2_ in air. During the analyses, the cuvette was continuously flushed with CO_2_‐free air at a rate ≥60 ml min^−1^ (Figure 2), and we allowed 2.5 min for cuvette equilibration before beginning measurements of exhaled CO_2_. Full equilibration was ensured by monitoring CO_2_ concentration in the cuvette and confirmed using segmented regression of CO_2_ concentration values. Because we suspected that in our 2010 attempt our measurements might not have been long enough for birds to achieve a fasting state, we took measurements for a longer period in 2018. For 17 of the 24 birds (1 Broad‐tailed male, 2 Broad‐tailed females, 3 Rufous males, and 11 Rufous females), the δ^13^C value of exhaled CO_2_ was measured at minutes 0 (2.5 min after the bird was sealed in the cuvette), 0.5, 1.5, 3.5, 7.5, 11.5, 15.5, and 19.5 (representing sampling intervals of 0.5, 1, 2 min, and repeated sampling at 4‐min intervals). The remaining 7 birds (5 Rufous males and 2 Rufous females) were held for up to 8 additional minutes. In these instances, measurements were also taken at minutes 23.5, 27.5, and 31.5. Birds were then removed from the cuvette, weighed, and offered a nectar meal using a 1 ml disposable plastic pipet filled with 25% weight/volume C_4_ cane sugar nectar. Prior to the release of each bird, we clipped a unique pattern of tail feathers. Reference gas ([CO_2_] = 21.630 ppm, δ^13^C = −9.9‰) readings were conducted every morning and evening for calibration and indicated a precision (standard deviation) of 0.1‰ and insignificant analytical drift.

Statistical analysis—Isotope values for each bird over time were estimated using a nonlinear model of the form:

$$\delta^{13}C_{t}=\delta^{13}C_{\infty }-\left(\delta^{13}C_{\infty }-\delta^{13}C_{0}\right)\;e^{-\frac{t}{\tau }},$$

where tis time, $\delta^{13}C$_0_ is the isotopic composition of exhaled CO_2_ in fed birds (minute 0), $\delta^{13}C$_∞_ is the asymptotic isotopic composition of exhaled CO_2_ in fasted birds, and τ is the average retention time in minutes (1/τ equals the instantaneous turnover; Martínez del Rio & Anderson‐Sprecher, 2008). As mentioned above, we use the terms fed and fasted to denote measurements taken on newly captured birds and birds for which the most recent meal has fully passed, respectively. Parameter estimation was conducted using a Bayesian framework that allowed us to estimate posterior distributions for $\delta^{13}C$_0_ and $\delta^{13}C$_∞_ individually for each bird, while simultaneously estimating a joint posterior distribution of τ for all^1^ birds from both species measured in 2010 and 2018 combined. Posterior distributions of individuals were pooled to infer population‐level distributions of δ^13^C_0_ and δ^13^C_∞_ for each species. After applying an isotope mixing model to estimate the contribution of the two nutrient resources to the population‐level pooled distributions, we were able to infer a distribution of the reliance feeder use in which the mode/peaks are more frequent estimated values. Uniform priors were used for all parameters with τ bound between 0 and 20 min, and $\delta^{13}C$_0_ and $\delta^{13}C$_∞_ bound between −10‰ and −30‰. These bounds reflect the maximum expected range in potential δ^13^C values of exhaled CO_2_ considering the δ^13^C values of potential dietary sources and accounting for diet‐to‐tissue discrimination (the difference in isotopic composition between a consumer's tissues and its diet resulting from preferential assimilation of one of the isotopic forms; Martinez del Rio et al., 2009) using values provided in Carleton et al. (2004, 2006). The Bayesian model was run over three Markov chain Monte Carlo chains, each consisting of 10,000 samples with the first 1000 removed and thinned by retaining every third sample. Convergence was confirmed by visual inspection of traceplots as well as ensuring the R‐hat diagnostic was <1.1 (Kery, 2010). All models were run in JAGS (ver. 4.1.0) using R (v. 3.4.0) as an interface. Differences in predicted δ^13^C values by species and between years were inferred by pooling the relevant posterior distributions of $\delta^{13}C$_0_ and $\delta^{13}C$_∞_ of individual birds. Results are presented as the modes and 85% highest posterior density intervals (hereafter, HPDIs). HPDIs are the narrowest interval that has the highest probability density and includes the mode of the estimated values. HPDIs are analogous to frequentist confidence intervals (Hespanhol et al., 2019). Distributions of feeder use were constructed by applying average diet‐to‐tissue discrimination values from Carleton et al. (2004, 2006) to a simple two‐component linear mixing model (e.g., Martinez del Rio et al., 2009) in which we generated expected values by varying the proportional contribution of C_4_ (feeder) sugars from 0% to 100%. Average diet‐to‐tissue discrimination values were applied as a simple addition to all posterior distribution values. Carleton et al. (2004, 2006)offer multiple diet‐to‐tissue discrimination values for fed (1.3‰, 1.8‰, and −1‰; Carleton et al., 2006) and fasted (2.9‰, 2.4‰, and 1.7‰; Carleton et al., 2006; and 0.6‰; Carleton et al., 2004) hummingbirds maintained on C_3_ and C_4_ diets. Given that we are not attempting to estimate the contributions of specific dietary resources at the scale of individual birds, but rather offer insight at population‐levels, we adopt the average of these diet‐to‐tissue discrimination values for fed ($\bar{x}$ = 0.7‰) and fasted ($\bar{x}$ = 1.9‰) birds.

## 3 RESULTS

Posterior distributions of τ indicate that birds switch metabolic fuel sources, from recently ingested sugars to stored resources (i.e., fed to fasted), within the measurement periods of both the 2010 and 2018 study designs (mode = 2.67 min, HPDI = 2.21–3.38 min; Table 1). This relatively short time validates our assumption regarding the effect of bout feeding on crop fullness and the consequent duration for which samples needed to be collected in order to adequately capture the switch from recently ingested exogenous fuel sources to endogenous stored lipid reserves.

**TABLE 1:** Eighty‐five percent highest posterior density intervals (HPDI) for the isotopic composition of exhaled CO_2_ in fed birds (δ^13^C_0_) and the isotopic composition of exhaled CO_2_ in fasted birds (δ^13^C_∞_) and mode and 85% highest posterior density intervals for the rate of change between δ^13^C_0_ and δ^13^C_∞_ (τ) for Broad‐tailed and Rufous hummingbirds

		τ (min)		$\delta^{13}C_{0}\left(‰\right)$	$\delta^{13}C_{\infty }\left(‰\right)$
		Mode	85% HPDI	85% HPDI	85% HPDI
*Selasphorus platycercus*	All Years	2.67	2.21–3.38	−24.5–−12.5	−25.0–−13.8
*Selasphorus rufus*	All Years	2.67	2.21–3.38	−21.2–−10.0	−26.5–−12.7
	2010	−	−	−23.3–−10.0	−25.8–−12.7
	2018	−	−	−19.5–−10.7	−27.3–−12.9

*Note*: The rate of change was within the mensuration time frame of both the 2010 and 2018 experiments. The 85% HPDIs for δ^13^C_0_ and δ^13^C_∞_ were similar between species and years.

**FIGURE 3:**
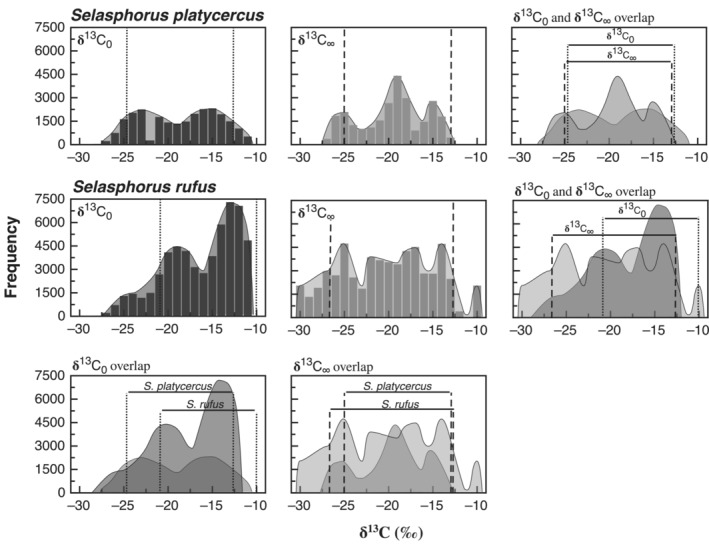
Posterior distributions for the isotopic composition of exhaled CO_2_ in fed birds (δ^13^C_0_; first column), the isotopic composition of exhaled CO_2_ in fasted birds (δ^13^C_∞_; second column), and associated overlap for Broad‐tailed and Rufous hummingbirds. Y axis values represent frequencies from 10,000 Markov chain Monte Carlo samples, with higher frequencies representing greater numbers of individuals likely to employ a particular dietary resource or mixture. Vertical dotted lines and vertical dashed lines represent the 85% highest posterior density intervals for δ^13^C_0_ and δ^13^C_∞_, respectively.

Posterior distributions of δ^13^C values for δ^13^C_0_ and δ^13^C_∞_ were wide‐ranging and showed variability within and between species. Generally, the HPDIs for δ^13^C_0_ and δ^13^C_∞_ spanned the range of expected δ^13^C values (Table 1, Figure 3). For example, HPDIs for δ^13^C_0_ in Broad‐tailed and Rufous hummingbirds were from −24.5‰ to −12.5‰ and −21.2‰ to −10.0‰, respectively (Table 1). For δ^13^C_∞_, HDPIs were −25.0‰ to −13.8‰ for Broad‐tailed hummingbirds and −26.5‰ to −12.7‰ for Rufous hummingbirds (Table 1). Despite these broad spreads, modal values, indicative of the most commonly used resource, were often different among distributions. In addition, several of the pooled distributions displayed multiple peaks (Figure 3), suggesting variable grouping in relative resource use among individuals. For Broad‐tailed hummingbirds, the posterior distribution for $\delta^{13}C$_0_ displayed peaks of near equal magnitude at approximately −23.0‰ and −15.0‰ (the peak at −15.0‰ had a slightly larger frequency). For $\delta^{13}C$_∞_, peaks were observed at approximately −25.0‰, −19.0‰, and −15.0‰, with the peak at −19.0‰ displaying a noticeably greater frequency (Figure 3). For Rufous hummingbirds, the posterior distribution for $\delta^{13}C$_0_ displayed peaks at approximately −19.0‰ and −12.0‰ with the peak at −12.0‰ displaying a noticeably greater frequency. For $\delta^{13}C$_∞_, peaks of near equal magnitude were observed at approximately −25.0‰, −17.0‰, and −14.0‰; although the peak at −25.0‰ had a slightly larger frequency than the others (Figure 3). We note that while these multiple peaks within distributions likely reflect commonly used mixtures of the two nutrient sources, the relatively small samples sizes and the analytical focus on individuals employed in our study may also contribute to this pattern. Comparison of HPDIs showed differences in the amounts of overlap between distributions (Figure 3), suggesting population‐level differences in resource use between species or fed vs. fasted states. For Broad‐tailed hummingbirds, HPDIs were similar for $\delta^{13}C$_0_ and $\delta^{13}C$_∞_, indicating substantial overlap in the range of isotopic values observed between the two distributions. In contrast, for Rufous hummingbirds, the HPDI for $\delta^{13}C$_0_ was both narrower and more enriched than that for $\delta^{13}C$_∞_. When comparing between species, Rufous hummingbirds had a more enriched, albeit similarly sized, HPDI for $\delta^{13}C$_0_ than Broad‐tailed hummingbirds and a wider HPDI for $\delta^{13}C$_∞_ that encompassed the $\delta^{13}C$_∞_ HPDI for Broad‐tailed hummingbirds (Figure 3).

**FIGURE 4:**
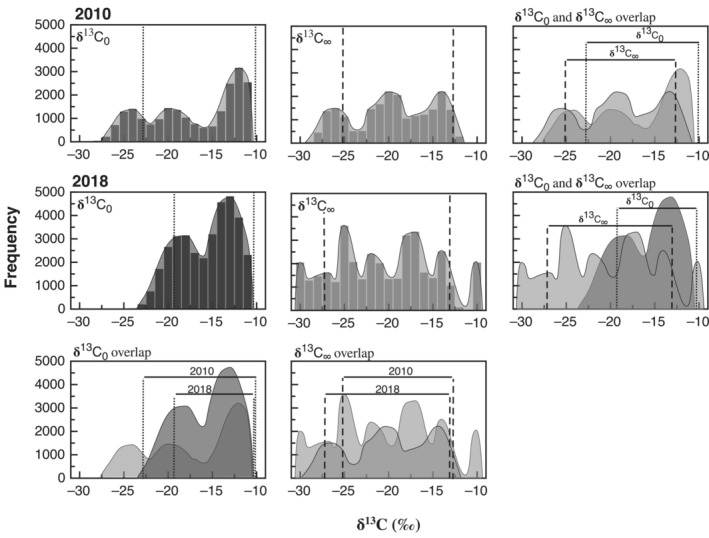
Posterior distributions for the isotopic composition of exhaled CO_2_ in fed birds (δ^13^C_0_; first column), the isotopic composition of exhaled CO_2_ in fasted birds (δ^13^C_∞_; second column), and associated overlap for Rufous hummingbirds in 2010 and 2018. Y axis values represent frequencies from 10,000 Markov chain Monte Carlo samples, with higher frequencies representing greater numbers of individuals likely to employ a particular dietary resource or mixture. Vertical dotted lines and vertical dashed lines represent the 85% highest posterior density intervals for δ^13^C_0_ and δ^13^C_∞_, respectively.

Comparison of posterior distributions between 2010 and 2018 showed differences between years. For $\delta^{13}$C_0_, HPDIs were −23.3‰ to −10.0‰ for Rufous in 2010 and −19.5‰ to −10.7‰ for Rufous in 2018. For $\delta^{13}$C_∞_, HPDIs were −25.8‰ to −12.7‰ for Rufous in 2010 and −27.3‰ to −12.9‰ for Rufous in 2018 (Table 1, Figure 4). Interannual comparisons were not attempted for Broad‐tailed hummingbirds because of the small sample size in 2018. Distributions for Rufous hummingbirds also exhibited multiple peaks. In 2010, the posterior distribution for $\delta^{13}C$_0_ displayed peaks at approximately −24.0‰, −20.0‰, and −12.0‰ with the peak at −12.0‰ displaying a noticeably greater frequency. For $\delta^{13}C$_∞_, peaks were observed at approximately −26.0‰, −20.0‰, and −14.0‰, with the peaks at −20.0‰ and −14.0‰ displaying nearly equal frequencies (Figure 4). In 2018, the posterior distribution for $\delta^{13}C$_0_ displayed peaks at approximately −18.0‰ and −13.0‰, with the peak at −13.0‰ displaying a noticeably greater frequency. For $\delta^{13}C$_∞_, main peaks were observed at approximately −25.0‰ and −17.0‰, with noticeably smaller sub‐peaks at approximately −22.0‰ and −14.0‰ (Figure 4). Again, comparison of HPDIs showed variability in the amounts of overlap between distributions (Figure 4). In both 2010 and 2018, HPDIs for $\delta^{13}C$_0_ were more enriched than those for $\delta^{13}C$_∞_. Further, in 2018, the HPDI for δ^13^C_0_ was substantially narrower than that for $\delta^{13}C$_∞_. Comparing between years, the HPDI for $\delta^{13}C$_0_ in 2010 was broader and encompassed more depleted values than that for 2018. The opposite was observed for $\delta^{13}C$_∞_, where the HPDI for 2018 was broader and encompassed more depleted values than that for 2010. We note; however, that both the methods and field sites differed between 2010 and 2018, and, as a result of these potentially confounding differences, disparities among years identified in this work should be viewed through the most conservative lens (e.g., compressed schedule on 2018 may have resulted in a lower proportion of birds that had accessed feeders).

**FIGURE 5:**
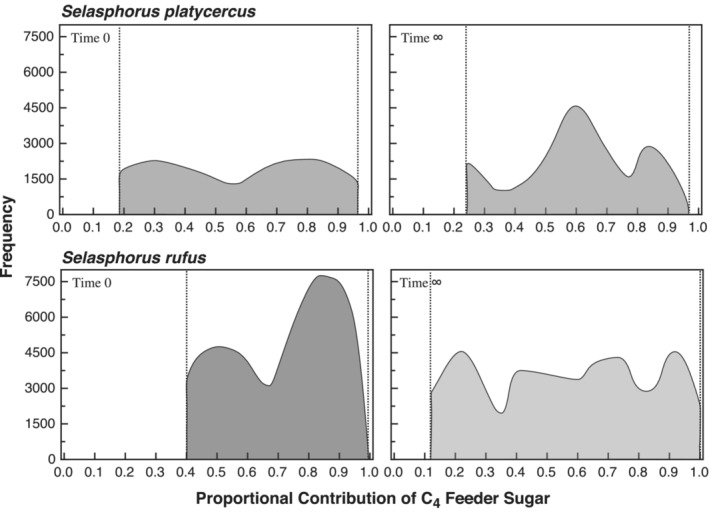
Estimated proportional contributions of feeder sugar to the diets of fed (δ^13^C_0_; first column) and fasted (δ^13^C_∞_; second column) Broad‐tailed and Rufous hummingbirds. Estimates are based on applying a simple two‐part mixing model and the discrimination values from Carleton et al. (2004, 2006) to the posterior distributions shown in Figure 2. Vertical dotted lines represent the 85% highest posterior density intervals. In general, both species use a mix of feeder and flower sugar to fuel metabolism, with Rufous hummingbirds demonstrating a greater use of feeders than Broad‐tailed hummingbirds.

Applying average discrimination values from Carleton et al. (2004, 2006) to a simple two‐part linear isotope mixing model (e.g. Martinez del Rio et al., 2009) in which we vary the proportional contribution of C_4_ (feeder) sugars from 0% to 100%, we see that when metabolism is fueled by recently ingested dietary resources, expected δ^13^C values for exhaled CO_2_ range from −27.3‰ to −12.1‰ (Figure 5). When metabolism is fueled by stored lipid reserves created using C_4_ (feeder) sugars, expected δ^13^C values for exhaled CO_2_ range from −28.5‰ to −13.3‰ (Figure 5). Comparison of these expected values to the 85% HPDIs generated by our analyses shows a broad range in the contribution of feeder sugars to fuel metabolism. For recently consumed sugars, comparison with the HPDI suggests proportional contributions of C_4_ feeder sugar ranging from 18% to 97% in Broad‐tailed hummingbirds, with peaks (i.e., most common values) at approximately 28% and 81%. In Rufous hummingbirds, estimated contributions ranged from 40% to 100%, with peaks at approximately 55% and 100% (Figure 5). When metabolism was fueled by stored reserves, the proportional contributions of C_4_ feeder sugar indicated by the HPDI ranged from 23% to 97% in Broad‐tailed hummingbirds, with peaks at approximately 23%, 62%, and 89%. For Rufous hummingbirds, the range of estimated contributions indicated by the HPDI was 13% to 100%, with peaks at approximately 23%, 76%, and 95% (Figure 4).

## 4 DISCUSSION

Our results suggest variability in the relative contributions of feeder and flower sugar to the metabolism of Broad‐tailed and Rufous hummingbirds. This variability is important in that it suggests that effects to both hummingbirds and the plants they pollinate resulting from the presence of feeders will not apply evenly within and across populations. Further, our results demonstrate the utility of using field measurements of δ^13^C in exhaled CO_2_ to examine resource use in nectar feeding birds. In the following sections, we consider both the use of exhaled CO_2_ in studying hummingbird energetic sources and the potential implications of our results.

### 4.1 Ecological implications of mixed resource use in hummingbirds

Our results suggest that when provided with feeders, Broad‐tailed and Rufous hummingbirds may adopt mixed resource use strategies, with many birds using resources from both feeders and flowers to fuel metabolism and in the creation of stored lipids. Further, we see evidence of differences in the frequencies with which different mixtures of the two dietary resources are used between species, time periods, and locations; with Rufous hummingbirds more likely to use a higher proportion of feeder sugar as both an exogenous fuel source and in the creation of stored lipids than Broad‐tailed hummingbirds (Figure 3). This was surprising, as we expected the strongly aggressive and territorial nature of Rufous hummingbirds (Calder & Healy, 2006; Hixon et al., 1983) to result in feeder guarding, functionally limiting the number of individuals that could access feeder sugar and resulting in high frequencies in stored lipids derived from nectar from wild flowers. In addition, both species exhibited more balanced use of feeder and flower sugars for lipid synthesis than was demonstrated for recently consumed dietary sugars (Figure 3). This is presumably, a function of differences in the availability of feeder sugar at the time of lipid synthesis compared to relatively liberal availability during our experiment. Regardless of its source, this variability has important implications on the potential ecological effects of hummingbird feeders. As hummingbirds serve as important pollinators of many plants (Arizmendi et al., 2007; Inouye et al., 1991; McCafferey & Wethington, 2008; Schuchmann, 1999), complete preference for feeder sugar would have potentially devastating effects on this symbiotic relationship and associated ecological effects between hummingbird populations and the flowers they pollinate. However, the intrapopulation variability in the use of feeder sugar demonstrated by our study may provide a buffer against these potential effects by limiting the degree to which flower nectar is supplanted by anthropogenic sources of sugar. While the specific mechanisms whereby this variability occurs are outside the scope of this study, we note that our experiments were conducted during the summer months in which numerous C_3_ flowers were in bloom, offering an ample food source for hummingbirds. In this regard, our results may reflect the relative availability of the two resource options and may parallel McCafferey and Wethington's (2008) observation of decreased feeder use with increased local flower abundance. Alternatively, in preference trials, hummingbirds have been observed to exhibit a marked preference for low volumes of higher sucrose concentration (>45%–50%) nectar that maximize their instantaneous energy intake rates (Blem et al., 2000; Roberts, 1996; Tamm & Gass, 1986), suggesting an optimal foraging strategy. While the sucrose solution used in the feeders in our study had a similar concentration to that of many hummingbird‐pollinated flower species (Baker, 1975), we did not conduct specific measurements of the sugar concentrations of local flowers and; thus, can offer neither direct comparison nor comment on how this may have influenced dietary resource use in our study. Further, despite mixed results from studies examining optimal foraging in hummingbirds (Hainsworth & Wolf, 1976; Montgomerie et al., 1984; Pyke, 1978, 1981), our study design did not allow for specific examinations of the potential relationships between resource use and feeder vs. flower abundance and density, competition, etc. Consequently, we recommend future field‐based research that applies the techniques described in the current study to explore the mechanisms leading to resource selection in hummingbirds.

Regardless of the specific mechanisms driving the patterns of resource use shown in our results, we stress that potential effects to individual birds of using anthropogenically supplied supplemental foods (e.g., increased exposure to predation and disease transmission; Robb et al., 2008) would not necessarily be mitigated by mixed resource use and may increase in risk with the frequency of feeder visits. For example, Miller and Gass (1985) report that the majority of recorded predation events on North American hummingbirds are at feeders found in flower gardens and feeders. This would, according to our results, put Rufous hummingbirds at greater individual risk than Broad‐tailed hummingbirds because of the greater frequency with which individual Rufous hummingbirds demonstrated high‐relative feeder use. However, we stress that the risks of predation, disease, and other possible negative effects of feeder use have received little research attention, are likely species and context dependent, and should be considered in balance with the potential positive effects of food supplementation, such as increased survival and reproductive success (Robb et al., 2008).

### 4.2 δ^13^C in exhaled CO_2_ as a dietary indicator

Our results clearly demonstrate the utility of using the carbon stable isotope value of exhaled CO_2_ to study resource use of nectar feeding birds in field settings. While this work is not the first to employ exhaled CO_2_ from bird breath to study dietary ecology and physiology, previous studies using this technique have largely been conducted in captive settings (e.g., Chen & Welch, 2014; Hatch et al., 2002; McCue & Welch, 2016; Voigt et al., 2008 and references therein). Relatively fewer studies have applied the measurement of δ^13^C in exhaled CO_2_ to study the diets of free‐living animals (e.g., McCue & Welch, 2016; Podelsak et al., 2005; Voigt, 2009; Voigt et al., 2010, 2013; Whiteman et al., 2012 and references therein). Potential reasons for the limited use of this technique in field settings are reflected in our work and may include the following: (1) technological limitations prior to the adoption of cavity ring‐down spectroscopy prevented real‐time measurements; thereby making the design and interpretation of field experiments difficult (i.e., were samples taken for a sufficiently long period?); (2) while vastly superior in size, portability, and expense to traditional mass spectrometers, cavity ring‐down spectroscopy units are still relatively bulky and cumbersome, thus limiting their deployment in field settings; (3) the potential for ambient CO_2_ to leak into the mensuration apparatus and contaminate samples (McCue & Welch, 2016) is higher in field settings where equipment may be repeatedly assembled and disassembled and moved frequently; (4) the single isotope value provided by exhaled CO_2_ may provide insufficient resolution for dietary studies in animals with multiple dietary sources. Despite these possible limitations, the successful field applications of breath δ^13^C measurements described in the aforementioned studies, and the significant potential of this technique as a rapidly deployable source for real‐time data, prompts us to urge its consideration for use in future studies. We do; however, note that for species, like hummingbirds, in which the change from using recently consumed sugars to stored lipids to fuel metabolism can occur very quickly, differences in the time between which the last meal was consumed, the bird is caught, and breath measurements commence may have substantial impacts to results. Consequently, we recommend that future studies conducted on these species consider paring δ^13^C measurements of exhaled breath with techniques such as respiratory quotient to determine whether current metabolism is fueled by carbohydrates or lipids.

## 5 CONCLUSION

Understanding the effects of providing hummingbirds with supplemental nectar from feeders is complicated by the logistical difficulties associated with traditional observational techniques. Our work, which refined existing methods to measure the δ^13^C in exhaled CO_2_, allowed us to assess the relative contributions of feeder and flower nectar to exogenous and endogenous metabolic fuel sources (1) without the need for direct observation of individual birds, and (2) in a manner that tracks the relative contributions of resources that are actually consumed and integrated, rather than simply visited.

Our results suggests that when provided with supplemental feeders, both Broad‐tailed and Rufous hummingbirds demonstrate great range in their use of feeder sugar. The Rufous hummingbirds in our experiment used higher levels of feeder sugar with greater frequency; however, both species adopted a mixed resource use strategy. While the specific mechanisms leading to the relative contributions of the two dietary sources to individual birds deserves further attention, the use of multiple energetic resources suggests resilience against the potential negative effects of anthropogenically supplied supplemental foods on hummingbirds and the plants they pollinate in wild settings.

## AUTHOR CONTRIBUTIONS

**Nathan Wolf:** Conceptualization (equal); data curation (equal); formal analysis (supporting); investigation (equal); methodology (equal); project administration (equal); resources (equal); visualization (equal); writing – original draft (equal); writing – review and editing (equal). **T Scott Smeltz:** Formal analysis (lead); writing – review and editing (equal). **Craig Cook:** Methodology (equal); resources (equal); visualization (equal); writing – review and editing (equal). **Carlos Martinez del Rio:** Conceptualization (equal); data curation (equal); formal analysis (supporting); investigation (equal); methodology (equal); visualization (equal); writing – original draft (equal); writing – review and editing (equal).

## CONFLICT OF INTEREST

The authors declare no conflict of interest.

## Data Availability

The data presented in this manuscript is available at the Dryad Data repository at https://doi.org/10.5061/dryad.1rn8pk0w8.

## Endnote

^1^
In 2018, two Rufous hummingbirds were retested after being fed sugars with contrasting δ^13^C values and were included when estimating τ. The retested birds were not; however, included in estimating any other covariates.
